# Climate Stressors on Growth, Yield, and Functional Biochemistry of two *Brassica* Species, Kale and Mustard

**DOI:** 10.3390/life12101546

**Published:** 2022-10-06

**Authors:** Akanksha Sehgal, Kambham Raja Reddy, Charles Hunt Walne, T. Casey Barickman, Skyler Brazel, Daryl Chastain, Wei Gao

**Affiliations:** 1 Department of Plant and Soil Sciences, Mississippi State University, P.O. Box 9555, Mississippi State, MS 39762, USA; 2 North Mississippi Research and Extension Center, P.O. Box 1690, Verona, MS 38879, USA; 3 USDA ARS Sustainable Water Management, 4006 Old Leland Road, Stoneville, MS 38756, USA; 4 USDA UVB Monitoring and Research Program, Natural Resource Ecology Laboratory, and Department of Ecosystem Science and Sustainability, Colorado State University, Fort Collins, CO 80523, USA

**Keywords:** temperature stress, elevated CO_2_, Ultraviolet (UV)-B, *Brassica oleracea*, *Brassica juncea*, chlorophyll, carotenoids

## Abstract

Due to climate change, the attainment of global food security is facing serious challenges in meeting the growing food demand. Abiotic stresses are the foremost limiting factors for agricultural productivity. However, not much information is available on the effect of multiple abiotic stresses on the morphological and biochemical aspects of kale and mustard. Therefore, an experiment was designed to study the effects of UV-B radiation, CO_2_ concentration, and high temperature on the growth, yield, and biochemistry of two *Brassica* species, namely *B. oleracea* L. var. acephala Winterbor F1 (hybrid kale) and B. *juncea* var. Green wave O.G. (mustard greens), which were grown under optimal nutrients and soil moisture conditions in soil–plant–atmosphere–research (SPAR) units. Two levels of UV-B radiation (0 and 10 kJ m^−2^ d^−1^), two concentrations of CO_2_ (420 and 720 ppm), and two different temperature treatments (25/17 °C and 35/27 °C) were imposed 12 days after sowing (DAS). Several morphological and biochemical parameters were measured at harvest (40 DAS) in both species. All the traits declined considerably under individual and multi-stress conditions in both species except under elevated CO_2_ levels, which had a positive impact. Marketable fresh weight decreased by 64% and 58% in kale and mustard plants, respectively, growing under UV-B treatment. A slight increase in the chlorophyll content was observed in both species under the UV-B treatment alone and in combination with high temperature and elevated CO_2_. Understanding the impacts of high temperature, CO_2_, and UV-B radiation treatments on leafy vegetables, such as kale and mustard, can help to improve existing varieties to enhance resilience towards environmental stresses while simultaneously improving yield, morphology, and biochemistry in plants.

## 1. Introduction

In the coming decades, a significant rise in agricultural productivity will be required to meet the food requirements of ~800 million undernourished people, which has been growing at an alarming pace, along with shrinking arable land [1,2,3]. In addition, adverse climate change has further exacerbated this, with increased abiotic stress conditions that detrimentally affect crop productivity and global ecosystem diversity [1,4,5,6]. Current temperatures are approximately 1 °C above pre-industrial levels, and a rise of 0.5 °C in global temperatures would increase the associated risks of high-temperature stress [7]. Furthermore, according to the fourth US climate assessment, a rise of 1.5 °C to 4.5 °C in global temperature has been projected in the next century due to an alarming increase in the levels of atmospheric CO_2_ and other greenhouse gases. The global atmospheric CO_2_ concentration is currently 417 ppm, as reported in March 2021 by the Mauna Loa observatory, Hawaii, while it was only 270 ppm during the pre-industrial era and is projected by climate models to reach 540 to 970 ppm by the year 2100 due to anthropogenic activities, reducing carbon sinks, and natural global cycles [8,9]. A substantial number of abiotic stress-related studies have been conducted during the past decade, but most of the experiments have focused on plants’ responses to individual stress treatment. The studies have overlooked the complex stress response generated in plants against combined or sequential abiotic stresses and the interaction of stresses with each other [10,11]. Therefore, it is imperative to understand the mechanisms associated with crops’ response to various abiotic stresses to manage future food production.

Kale (*Brassica oleracea* L. var. *acephala*.) and mustard (*Brassica juncea* L.) are leafy green brassica vegetables that have increased in production over the past ten years in response to demand by North American consumers. Both vegetables are considered highly nutritious leafy green vegetables even though their nutrient profile has not been well characterized to date [12]. Kale and mustard are rich sources of dietary fiber and low molecular weight nondigestible carbohydrates (LMWC), as well as vitamins A, K, and C; they also contain the essential minerals potassium (K), calcium (Ca), and magnesium (Mg). Despite the nutritional benefits, along with significant quantities of carotenoids and folates, none of these nutritional quality traits have been well characterized to date [13]. A recent study revealed that kale grown in the Southern US has the potential to provide significant quantities of several essential minerals and adequate quantities of LMWC, also known as “prebiotic carbohydrates”, with moderate to low levels of protein and energy [12]. 

Due to the suitability of kale for southern fall and winter growing conditions, kale has become a significant *Brassica* vegetable crop in the Southern US. Indeed, the Southern US has emerged as a leading kale production region over the last five years, responsible for more than two-thirds of the US crop [14]. Kale production in southern areas of the US has been increasing to meet American consumer demand, which has been growing at near-doubling rates in recent years [15]. Although kale production has been increasing, *Brassica* vegetables are generally widely under-consumed by Americans; this has been acknowledged in the current Dietary Guidelines Advisory Committee, which calls for Americans to increase their consumption of fruits and vegetables [15]. Both kale and mustard are hardy, cool season crop that tolerates summer heat but grows best in the fall and winter in the southern regions of the US. The suitable growth temperature is 15–22 °C [16,17]. However, kale and mustard are sensitive to high temperatures [18]. Thus, elevated temperatures and high UV-B levels can damage growth and developmental processes. Since the abiotic stresses are interlinked, the combination of their effects on the morphological, physiological, and cellular processes results in various changes in plant growth, productivity, and yield. Understanding plants’ mechanisms in response to multiple abiotic stresses are essential in devising management and breeding decisions in the near future.

Deryng et al. [19] contributed significantly to the current understanding of climate change impacts on crops under high temperatures and elevated CO_2_ concentration. Heat stress leads to poor germination and plant establishment, reduced photosynthesis, leaf senescence, decreased pollen viability, and fewer grains with smaller grain sizes [20,21]. It has been revealed in previous studies that a significant direct effect of increased CO_2_ on plant growth and yield could be seen that can compensate for a potentially hotter climate. Although increased CO_2_ concentrations have been reported to significantly increase yield in C_3_ plants [22,23,24], not many direct effects have been recorded on kale and mustard plants. The projected higher doses of incoming UV-B radiation can stimulate a variety of responses in higher plants [25,26]. Some harmful effects of UV-B radiation on plants include DNA damage, dilation, and disintegration of cellular membranes, photooxidation of leaf pigments and phytohormones, and inhibition of photosynthesis [27,28,29]. Additionally, UV-B radiation leads to changes in leaf thickness, anatomy, and canopy morphology, eventually affecting photosynthesis in plants [30]. Therefore, to enhance the production of green leafy vegetables with proper management and breeding strategies in the coming years, it is crucial to understand the effects of UV-B radiation individually and its interaction with other stresses. While some data exist on the crop’s growth, development, and productivity in response to individual CO_2_, temperature, or UV-B stresses, very little data is available on the effects of the interaction of these multiple factors on the growth and development of kale and mustard.

Field studies for understanding the interactive effect of abiotic stresses on *Brassica* spp. are tedious, discrepant, and seasonally limited. Therefore, simple, rapid, and reliable techniques are required to understand the response of these crops to various environmental stresses. The present study’s results can help to quantify *Brassica* species’ response to these abiotic stresses and improve existing varieties for enhanced resilience while improving the plants’ yield, morphology, and biochemistry. Compared to other controlled environment facilities, the soil–plant–atmosphere–research (SPAR) systems have the advantage of precisely controlling air temperature, CO_2_ concentration, UV-B dosage, and air humidity under natural solar radiation compared to other controlled environment facilities [31].

Since only limited information is available, the present study was designed to evaluate the interactive effects of elevated CO_2_, high temperature, and UV-B stress on kale and mustard plants’ morphology, physiology, and phytochemistry.

## 2. Materials and Methods

### 2.1. Experimental Conditions and Plant Material

This study included two *Brassica* species, namely B. *oleracea* var. *acephala* Winterbor F1 (hybrid kale) and B. *juncea* var. Green Wave OG (mustard greens). The experiment was conducted in a controlled environment facility (SPAR units) at the Environmental Plant Physiology Laboratory, Mississippi State University (33°28′ N, 88°47′ W), Mississippi State, MS, July–August 2019. The specifications and operation of SPAR units have been discussed in detail in Reddy et al. [32].

Seeds of the two *Brassica* sp. were sown in 30.5 by 15.2 cm (height by diameter) polyvinyl chloride pots filled with a soil medium consisting of 3:1 sand/topsoil (*v/v*). Before the start of treatments, the seedlings were thinned down to one plant per pot. The plants were watered and fertilized with a full-strength Hoagland nutrient solution [33] based on daily evapotranspiration (Table 1). Pots were arranged in 10 rows with three pots per row in each SPAR chamber with alternating rows of kale and mustard plants. The experiment consisted of 2 factors (8 levels of treatments × 2 species) with 15 replications. The pots were randomly arranged within each SPAR unit to avoid positional effects. In this study, 240 plants (2 species × 8 treatments × 15 replications) were used to estimate the impact of multiple stresses on the two *Brassica* species.

### 2.2. Treatments

The treatments included combinations of two [CO_2_] concentrations, namely 400 and 720 μmol mol^−1^ (+CO_2_), two different temperatures, namely 25/17 °C and 35/27 °C (+T) (day/night)], and two daily biologically effective UV-B radiation intensities, namely 0 and 10 kJ m^−2^ d^−1^ (+UV-B).

The control treatment was 400 μmol mol^−1^ [CO_2_], at 25/17 °C (day/night) temperature, and 0 kJ m^−2^ d^−1^ UV-B treatment. All SPAR chambers were maintained at control conditions until 12 days after sowing (DAS). Subsequently, each chamber was set at one of the following eight treatments until the final harvest (40 DAS): (1) a control treatment with optimum temperature, ambient CO_2_ levels, and no UV-B; (2) optimum temperature with elevated CO_2_ levels and no UV-B (+CO_2_); (3) elevated temperature with ambient CO_2_ levels and no UV-B (+T); (4) optimum temperature and ambient CO_2_ levels with 10 kJ UV-B (+UV-B); (5) elevated temperature and CO_2_ levels with no UV-B (+T + CO_2_); (6) optimum temperature with elevated CO_2_ levels and 10 kJ UV-B (+CO_2_ + UV-B); (7) elevated temperature with 10 kJ UV-B at ambient CO_2_ levels (+T + UV-B); (8) elevated temperature and elevated CO_2_ levels with 10 kJ UV-B (+UV-B + CO_2_ + T). The set and measured environmental variables in this study using eight different SPAR units are provided in Table 1.

### 2.3. Measurements

#### 2.3.1. Morphological Measurements

At 40 DAS, kale and mustard plants from each SPAR unit were hand-harvested to obtain their phenotype and growth data on the effects of multiple abiotic stresses. Plant height (PH, cm) was measured, leaf number (LN) was counted, and then total leaf area (LA, cm^2^ plant^−1^) was determined using an LI-3100 leaf area meter (LI-COR, Lincoln, NE, USA). Plant components, such as aboveground dry weights and root weights (g plant^−1^), were determined by drying the samples at 80 °C until a constant weight was reached.

#### 2.3.2. Physiological Measurements

Leaf chlorophyll content, epidermal flavonoids index, epidermal anthocyanin, and nitrogen balance index (the ratio of chlorophyll content/flavonoids) were measured on the uppermost, fully expanded leaf, second from the top, across all treatments using a handheld Dualex Scientific instrument (Force A DX16641, Paris, France) at 35 DAS.

#### 2.3.3. Epicuticular Wax Content Determination

The extraction and quantitative analysis of leaf epicuticular waxes were carried out as per the method of Ebercon et al. [34] with minor modifications. Ten leaf discs constituting an area of 35.36 cm^−2^ from the third or fourth leaf from the stem apex were cut from both species from five plants in each treatment. Leaf discs were stirred in 15 mL of chloroform (Sigma-Aldrich, Inc., St. Louis, MO, USA) in a test tube for 20 s to remove leaf waxes. The wax extract was evaporated on a water bath maintained at 80 °C, and then cooled to room temperature; 5 mL of dichromate reagent was added and heated on a water bath held at 80 °C for 30 min. The samples were removed from the water bath and cooled, followed by the addition of 12 mL of de-ionized water. The samples were then allowed to stand for 15 min. The intensity of the color was measured at 590 nm using a Bio-Rad UV/VIS spectrophotometer (Bio-Rad Laboratories, Hercules, CA, USA). The wax content was expressed on a leaf area basis (μg cm^−2^) using a standard curve developed from the wax obtained from the same species.

#### 2.3.4. Carotenoid Analysis

Carotenoid pigments were extracted and analyzed from freeze-dried leaf tissues, according to Kopsell et al. [35,36], with a few changes, as described by Barickman et al. [37].

### 2.4. Data Analysis

#### 2.4.1. Combined Stress Response Index (CSRI)

Based on the summation of relative individual stress responses at each treatment and similar to the cumulative response index quoted in other UV-B studies [38], the combined stress response index (CSRI) was calculated to evaluate the interactive effects of eight treatments (+ CO_2_, + T, + UV-B, + CO_2_ + T, + CO_2_ + UV-B, + UV-B + T, and + CO_2_ + T + UV-B) in comparison to control treatment. The CSRI was calculated as the value of a parameter under control (c) subtracted from the value of the parameter under treatment (t), and then by dividing from the value of a parameter under control (c) as follows:

$$\begin{array}{c} \mathrm{CSRI}=\frac{\left(PHt-PHc\right)}{\left(PHc\right)}+\frac{\left(LNt-LNc\right)}{\left(LNc\right)}+\frac{\left(LAt-LAc\right)}{\left(LAc\right)}+\frac{\left(MFWt-MFWc\right)}{\left(MFWc\right)}+\frac{\left(ADWt-ADWc\right)}{\left(ADWc\right)}+\\ \frac{\left(RDWt-RDWc\right)}{\left(RDWc\right)}+\frac{\left(TDWt-TDWc\right)}{\left(TDWc\right)}+\frac{\left(RSt-RSc\right)}{\left(RSc\right)}+\frac{\left(Neot-Neoc\right)}{\left(Neoc\right)}+\frac{\left(Violt-Violc\right)}{\left(Violc\right)}+\\ \frac{\left(Zeat-Zeac\right)}{\left(Zeac\right)}+\;\frac{\left(Lutt-Lutc\right)}{\left(Lutc\right)}+\frac{\left(Bcart-Bcarc\right)}{\left(Bcarc\right)}+\frac{\left(TXant-TXanc\right)}{\left(TXanFc\right)}+\;\frac{\left(ZA/ZAVt-ZA/ZAVc\right)}{\left(ZA/ZAVc\right)}+\\ \frac{\left(Chlt-Chlc\right)}{\left(Chlc\right)}+\frac{\left(Flavt-Flavc\right)}{\left(Flavc\right)}+\frac{\left(Antht-Anthc\right)}{\left(Anthc\right)}+\frac{\left(NBIt-NBIc\right)}{\left(NBIc\right)}+\frac{\left(Waxt-Waxc\right)}{\left(Waxc\right)} \end{array}$$

Here, CSRI is the combined stress response index, PH is the plant height, LN is the leaf number, LA is the leaf area of the plant, MFW is the marketable fresh weight, ADW is the aboveground dry weight, RDW is the root dry weight, TDW is the total dry weight, RS is the root-to-shoot ratio, Neo is the neoxanthin concentration, Viol is the violaxanthin concentration, Zea is the zeaxanthin concentration, Lut is the lutein concentration, Bcar is the β-carotene concentration, TXan is the total xanthophyll concentration, ZA/ZAV is the xanthophyll cycle ratio, Chl is the chlorophyll concentration, Flav is the flavonoid index, Anth is the anthocyanin index, NBI is the nitrogen balance index, Wax is the wax content, under t (treatment) and c (control).

#### 2.4.2. Statistical Analysis

The experimental layout was a split plot with a complete randomized block design, considering multi-stress treatments as the whole plot and species as the subplot. The one-way ANOVA of the general linear model, PROC GLM, was performed to test the effects of treatments, species, and their interactions on the measured traits using SAS 9.2 (SAS Institute, Cary, NC, USA). Fisher’s protected least significant difference tests at *p* = 0.05 were employed to test the differences among treatments for measured parameters. The standard errors of the mean were calculated and presented in the figures as error bars. Graphs were generated using Sigma Plot 13.0 (Systat Software, San Jose, CA, USA).

## 3. Results

### 3.1. Aboveground Morphology Parameters

**Figure 1:**
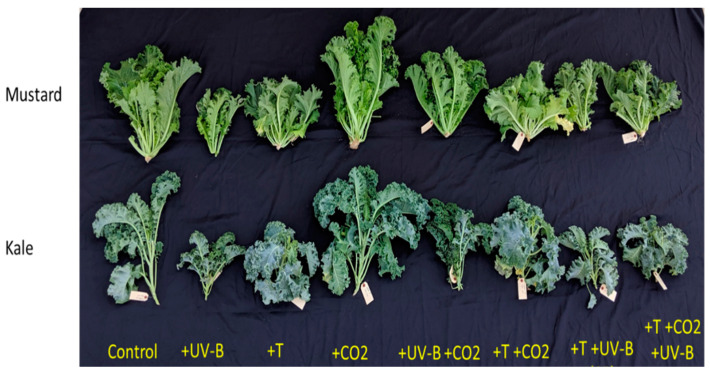
Pictorial representation of climate stress factors either alone or in combination on kale and mustard growth and development, 40 days after sowing, as follows: control, ultraviolet-B radiation (+UV-B), high temperature (+T), and elevated carbon dioxide (+CO_2_), ultraviolet-b radiation and elevated CO_2_ (+Uv-B + CO_2_), high temperature and elevated CO_2_ (+T + CO_2_), high temperature and high ultraviolet- radiation (+T + UV-B), and all three stressors combined (+T + CO_2_ + UV-B).

Here, UV-B and mostly elevated temperatures reduced vegetative growth in both crops. However, CO_2_ masked most of the other stresses’ adverse effects. Treatment and species interaction significantly affected the plant height, number of leaves, leaf area, and marketable fresh weight parameters (Table 2; Figure 1). Plants grown under +UV-B + T treatment were significantly shorter in both Brassica sp. compared to the control plants.

Elevated CO_2_ (+CO_2_) slightly increased plant height (7%) in kale and mustard compared to the control treatment. The highest reduction in plant height (47%) was observed in kale plants growing under +UV-B treatment and +T + CO_2_ (30.7%) for mustard. Mustard had taller plants and fewer reductions among the two species (Table 3).

The two *Brassica* species exhibited different responses for the number of leaves produced, among other treatments. A maximum reduction of 35% in the mustard leaves was observed under the +UV-B treatment, whereas the leaf number in kale remained unaffected (Table 3). Elevated CO_2_ treatment (+CO_2_) significantly increased the leaf number in both species. The highest number of leaves in mustard was found under +T + CO_2_ treatment and +UV-B + CO_2_ treatment in kale plants. Leaf number under the combination of all three stresses (+UV-B + CO_2_ + T) decreased by 1.5% in kale, whereas an increase of +14.8% was observed in mustard.

**Figure 2:**
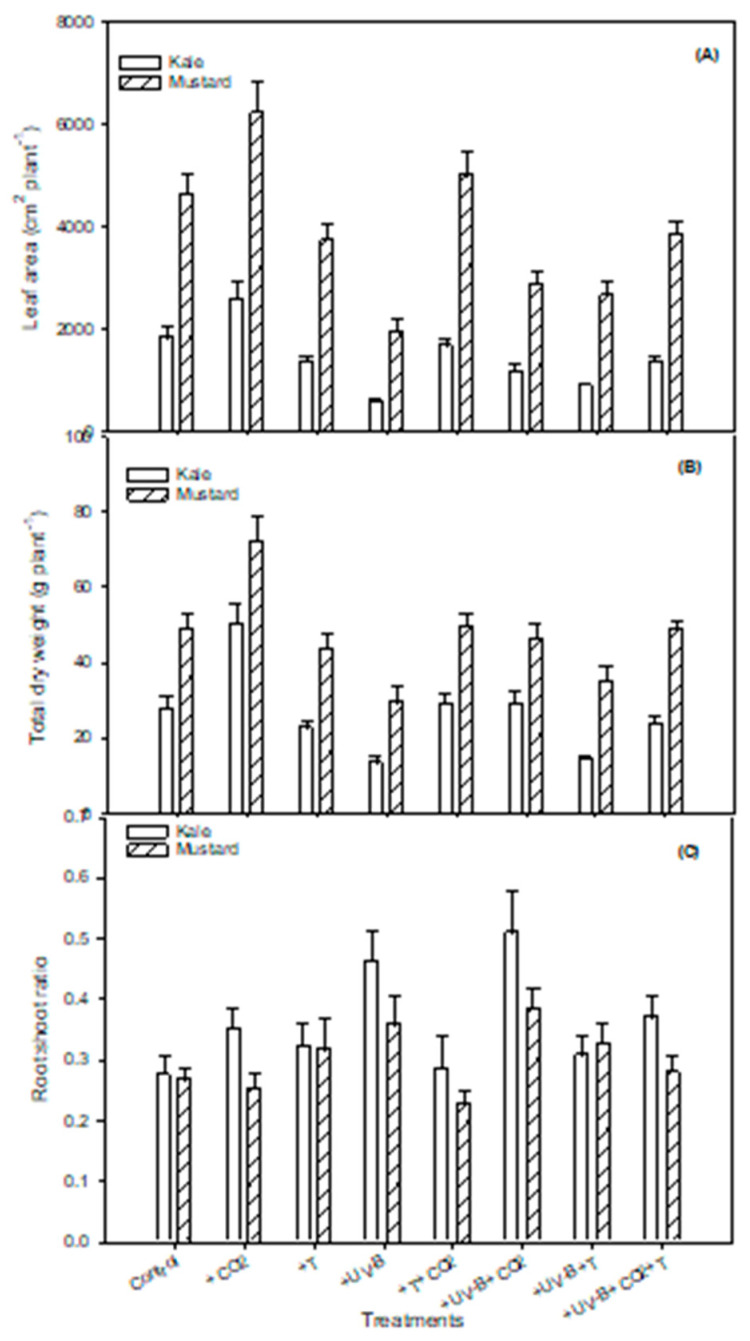
Impact of CO_2_ concentration (control, 400 μmol mol^−1^ and + CO_2_, 720 μmol mol^−1^), elevated temperatures (25/17 °C and 35/27 °C (day/night)), and UV-B radiation (control, 0 and + UV-B, 10 kJ m^−2^ d^−1^), and their interactions on (**A**) leaf area, (**B**) total dry weight, and (**C**) root/shoot ratio for kale and mustard. Bars indicate standard errors of the mean.

Even though +UV-B + CO_2_ treatment did not alleviate the negative effect of UV-B on leaf area, +CO_2_ treatment alone or in combination with high-temperature treatment (+T + CO_2_) increased leaf area by 39% and 2% in kale and 34.8% and 8% in mustard, respectively, in comparison to the control (Table 3, Figure 2A). Maximum reduction in leaf area was observed under UV-B treatment, with 68.4% (kale) and 58% (mustard) compared to the control. Mustard plants showed higher leaf area values than kale for all treatments (Figure 2A).

**Figure 3:**
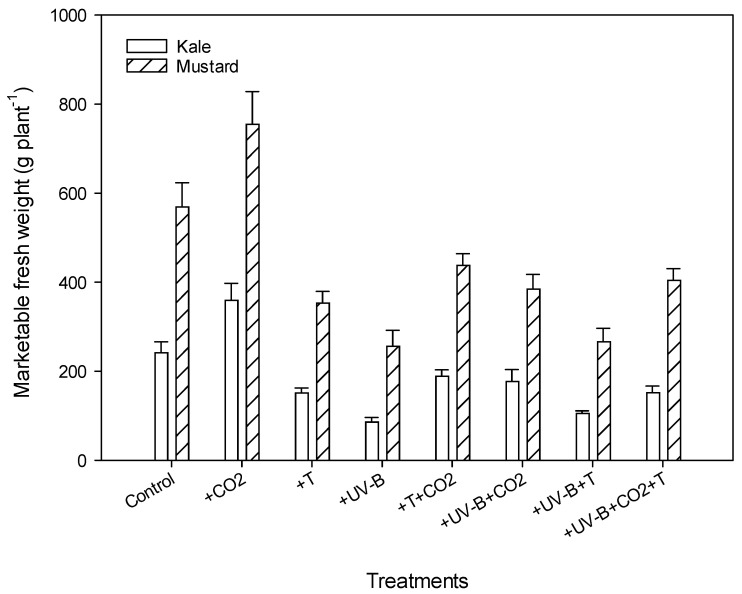
Impact of CO_2_ concentration (control, 400 μmol mol^−1^ and + CO_2_, 720 μmol mol^−1^), elevated temperatures (25/17 °C and 35/27 °C (day/night)), and UV-B radiation (control, 0 and + UV-B, 10 kJ m^−2^ d^−1^), and their interactions on marketable fresh weight for kale and mustard. Bars indicate standard errors of the mean.

A significant decrease in marketable fresh weight was observed under all treatments except +CO_2_. The UV-B treatment alone (+UV-B) showed a 64% and 58% decrease in fresh weight in kale and mustard, respectively. In contrast, the +UV-B + T treatment exhibited a 56% (kale) and 53% (mustard) decrease compared to the control condition (Figure 3). Maximum values for fresh weight were recorded under +CO_2_ in kale and mustard compared to their control showed a 49% and 32.6% increase.

### 3.2. Dry Weight Components

All the dry weight components displayed insignificant differences under the treatments–crop interaction (Table 2). Aboveground dry weight, root dry weight, and the total dry weight (Figure 2B) increased under +CO_2_ treatment, whereas they decreased to the lowest under +UV-B treatment in both kale and mustard (Table 3). Under +CO_2_ treatment, 78% and 48% more dry matter was produced in kale and mustard, respectively, compared to their control counterparts. High temperature, either alone (+T) or in combination with UV-B (+UV-B + T), also showed a considerable reduction in dry weight components (Table 3). Root/shoot ratio increased under all the treatments, but a maximum increase was observed under +UV-B treatment in both kale and mustard (Figure 2C, Table 3).

### 3.3. Physiological Parameters

**Figure 4:**
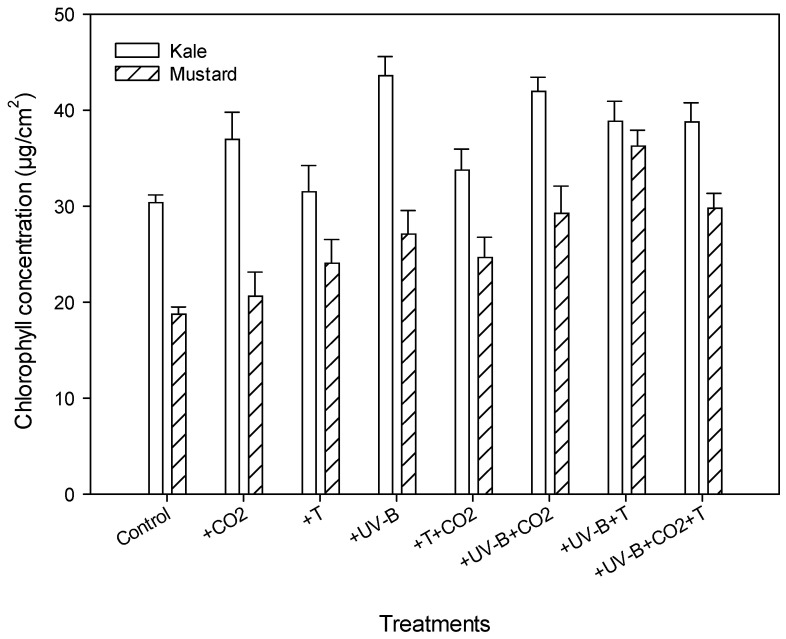
Impact of CO_2_ concentration (control, 400 μmol mol^−1^ and + CO_2_, 720 μmol mol^−1^), elevated temperatures (25/17 °C and 35/27 °C (day/night)), and UV-B radiation (control, 0 and + UV-B, 10 kJ m^−2^ d^−1^), and their interactions on chlorophyll concentration for kale and mustard. Bars indicate standard errors of the mean.

The different treatments and species affected the chlorophyll content, flavonoid index, anthocyanin index, and nitrogen balance index (Table 4). The interaction of treatment and crop for all four parameters was significant (Table 2). Chlorophyll content and flavonoid index increased, whereas anthocyanin index decreased under all treatments in both species. The chlorophyll content showed a maximum increase of 43.4% under +UV-B treatment in kale and 93.3% under +UV-B + T treatment in mustard (Figure 4, Table 4). The average flavonoid index ranged from 0.72–1.40, showing the highest increase under the +UV-B + CO_2_ + T treatment and a minimum increase under +T alone in kale and mustard.

The minimum adverse effect on the anthocyanin index was observed under +T treatment for kale and +CO_2_ treatment for mustard, with an average decrease of 11% and 7%, respectively, compared to the control. There was no change observed in the anthocyanin index under +CO_2_ treatment alone and in combination with high temperature (+T + CO_2_) in kale. Additionally, UV-B treatment alone (+UV-B) and in combination with CO_2_ and T (+UV-B + CO_2_, +UV-B + T, +UV-B + CO_2_ + T) showed significant reductions in the anthocyanin index (Table 4).

Nitrogen balance index (NBI) increased under all treatments in mustard except under +UV-B + CO_2_ + T, where a 6% decrease was observed compared to the control. A maximum decrease of 30% was observed in kale under +UV-B + CO_2_ + T, whereas an increase in NBI was exhibited under +CO_2_, +T, and their combination (Table 4).

### 3.4. Epicuticular Wax Content

Wax production was significantly increased under +T + CO_2_ treatment for kale and +UV-B+ CO_2_ treatment for mustard; the increase was 15% and 20.6%, respectively, compared to their control treatment (Table 4). The average wax content ranged from 66 to 140.8 μg cm^−2^ in kale and 21 to 11 μg cm^−2^ in mustard leaves. The lowest epicuticular wax was recorded under +UV-B + CO_2_ treatment in kale and under UV-B + CO_2_ treatment in mustard (Table 4). Kale plants grown under +UV-B + T + CO_2_ treatment and mustard plants grown under +UV-B treatment produced less wax on the leaves, and the reduction was 42.7% and 30%, respectively, compared to the plants growing under control conditions. Kale plants had the highest wax content under all the treatments.

### 3.5. Total Carotenoid Concentration

None of the pigments displayed significant differences under the treatments–crop interaction (Table 2); however, a substantial reduction in ZA/ZV in kale (38.3%) was observed under +UV-B + CO_2_ treatment and in mustard (19%) under +UV-B + T treatment compared to their respective control. Total xanthophyll content increased highest in kale under +UV-B + CO_2_ treatment, whereas a decrease of 10% was observed in mustard under the same treatment. An increase of 19% was recorded in total xanthophylls in mustard plants at elevated CO_2_ concentrations. Furthermore, β-carotene was atypically much lower (20%) under elevated CO_2_ in mustard than in control plants (Table 5). Neoxanthin, violaxanthin, zeaxanthin, lutein, and β-carotene increased significantly in kale plants at high CO_2_ concentrations (Table 5). Under +UV-B treatment, both Brassica species increased the concentration of neoxanthin, violaxanthin, and lutein.

### 3.6. Combined Stress Response Index (CSRI)

**Figure 5:**
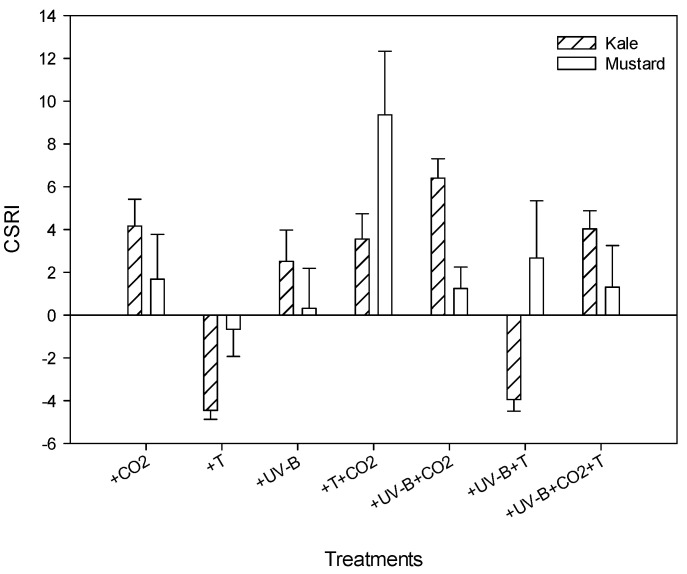
Cumulative stress response index (CSRI) was calculated over all the treatments of kale and mustard in response to elevated carbon dioxide (720 ppm) (+CO_2_), high temperature (35/27 °C day/night) (+T), and increased UV-B radiation (10 kJ m^−2^ d^−1^) (+UV-B) and their interactions.

The CSRI values ranged from −4 to 6.4 in kale and −0.6 to 9.3 in mustard. The lowest CSRI value for both crops was observed under the +T treatment. The highest CSRI value for kale was observed under +UV-B + CO_2_ treatment, whereas for mustard it was observed under +T + CO_2_ treatment. The CSRI values under all the treatments except +T treatment were positive in mustard. However, CSRI values under +T treatment and its combination with high UV-B levels (+UV-B + T) were negative for kale (Figure 5).

## 4. Discussion

*Brassica* plants are often exposed to multiple stresses concurrently during their growing season. Thus, when experimenting with growth chambers, it is essential to conduct experiments in an environment that mimics the natural conditions. This experiment studied two *Brassica* sp. for their response of morphological and physiological parameters to high temperature, UV-B treatment, and high CO_2_ and their interactions. To the best of our knowledge, this is the first study that tests leafy *Brassica* species across various abiotic stresses in combination.

The recent reduction in plant height under higher UV-B levels, like our study, has been reported in *Capsicum annuum* [39,40] and *Brassica napus* [41]. Reduced height in plants exposed to UV-B radiation implied that the specific photomorphogenic response of plants could be related to a UV-B photoreceptor by UV-B radiation [42]. Moreover, to some extent, low photosynthetically active radiation (PAR, 400–700 nm) might have also affected plant height. Colett et al. [43] reported that increased PAR reduces the impacts of UV-B radiation on plant height [43]. In another study conducted by Conner and Zangori [44] with two other *Brassica* species (*B*. *rapa* and *B*. *nigra*), reduced plant height was observed in plants exposed to high UV-B radiation [44].

Heat stress decreases stem growth by reducing cell size through the loss of cell water content, resulting in reduced plant height [45,46,47]. Following our results, many crops have reported reduced plant height at higher temperatures, including recent reports in Brassica juncea [48] and Oryza sativa [49]. Heat stress mainly affects the plant meristems, promotes leaf senescence and abscission, and reduces photosynthesis, ultimately reducing plant growth [50,51]. The number of leaves was decreased in *Brassica napus* under high UV-B levels [52] and at high temperatures [53]. Another study, under controlled conditions, showed a decrease in leaf number in different quinoa (Chenopodium quinoa Willd.) varieties under elevated UV-B radiation levels [54,55], further confirming our results.

Raghuvanshi and Sharma [56] suggested that decreased concentration of photosynthetic pigments was associated with a decline of leaf area in *Phaseolus vulgaris* under UV-B treatment, which further resulted in reduced growth, stem length, and root dry weight. These factors can lead to lower absorption of sunlight and affect photosynthetic activity, leading to a decrease in photosynthesis and indirectly affecting plant growth. The UV-B radiations mainly reduce cell division and expansion, reducing leaf area [56,57,58]. In contrast, Nedunchezhian and Kulandaivelu [59] observed in cowpea that slightly elevated UV-B radiation increases leaf area i.

The decrease in marketable fresh weight under UV-B treatment in the present study is supported by Cechin et al. [60], who reported that the decline in fresh weight production in crops is predominately due to UV-B exposure. Plants exposed to UV-B might allocate more energy for other physiological activities, especially defense mechanisms, rather than biomass production, resulting in a UV-B-mediated synthesis of protective pigments, such as carotenoids, anthocyanins, or phenolic acids [61,62]. The increased CO_2_ through increased activity of rubisco enzyme and reduced photorespiration increases leaf photosynthesis inside the leaf, leading to increased fresh weight and dry weight [63].

Results observed in *Arabidopsis thaliana* leaves [64], soybean [65], cotton [25], maize [29,66]; *Phaseolus vulgaris* [67], sweet potato [68], and basil [69] further corroborated our results, such as a reduction in total leaf area and fresh and dry weights, number of leaves, and height of plants (Table 3) under higher UV-B levels. In contrast to our results, Sakalauskaite et al. [70] reported an increase in plant height, leaf area, and dry weight under elevated UV-B in *Ocimum basilicum*. Reduction in leaf area in rice plants under high temperature alone and combination with elevated CO_2_ concentration has recently been reported by Wang et al. [71]. High temperature and UV-B interaction decreased leaf area in *Brassic napus* [41]. A decrease in fresh and dry weight was reported in *B. campesteris* under high-temperature conditions [72].

Our study’s dry weight reduction can be explained as a reaction to stress caused by UV-B radiation in plant development and metabolism [73]. In *Beta vulgaris*, a decrease of 10–12% in dry weight was reported under high UV-B levels [74]. On the contrary, increased dry weight was observed under high UV-B levels in broad bean and wheat plants [75]. Similarly, studies on broad bean and wheat, *Prunella vulgaris* plants, when exposed to 15-day UV-B radiations in a growth chamber, showed an increase in whole plant dry weight [76]. This suggests that the UV-B effect is species/cultivar specific, and sometimes it benefits the growth and development of some crops [77]. Indeed, UV-B exposure led to a significant decrease in root weight which could have caused an increased shoot/root ratio. Similar results under high UV-B levels were observed in *Manihot esculentum* [78] and under +CO_2_ levels in *Raphanus sativus* and *Daucus carota* [79].

Reduced plant weight under high temperatures can also be related to decreased photosynthesis, increased transpiration [80], and, in turn, reduced water use efficiency (WUE) [81]. A decrease in dry weight has been recently reported in three *Brassica* sp. [82], namely *Brassica oleracea* [83], *Raphanus sativus* [84], and *Chenopodium quinoa* [85], which further validates our study. High temperature and UV-B interaction decreased leaf weight in *Brassica napus* [41]. Mustard plants, compared to kale, showed higher dry weight under all the treatments. The concentration of CO_2_ lessened the effects of high temperature and UV-B, resulting in lesser reductions. Interaction of high temperatures and elevated CO_2_ increased plant height, the number of leaves, and leaf area in Fragaria x ananassa [86], *Capsicum annuum* [87], and *Solanum lycopersicum* [88]. In contrast to our results, Wang et al. [71] reported reduced whole plant dry weight in rice under high temperatures and elevated CO_2_ [71].

An increase in chlorophyll content under high temperatures has also been recently reported in other vegetable crops, such as tomatoes [89] and basil [90]. A recent study reported increased flavonoid content in kale under high UV-B levels [91], confirming our findings. Due to the strong antioxidant activity that flavonoids possess, higher total flavonoid content in the leaf implies a higher nutritional value in leaves [92]. Our results are consistent with the results of a study conducted by Olsson et al. [93], which reported a 70–150% increase in the overall flavonoid content of *B. napus* when subjected to high UV-B levels. Analogous to our results, an increase in anthocyanin content was reported in *Ocimum basilicum* under elevated UV-B levels by Sakalauskaite et al. [70]. The nitrogen balance index (NBI) is one of the critical indicators for crop growth. The NBI indicates C/N allocation changes due to N deficiency [94,95,96]. An increase in NBI was also observed in basil [90] and canola [97] under high temperatures.

A higher amount of wax for those leaves that were developed under elevated CO_2_ with UV-B radiation might have reduced the amount of incident UV-B radiation penetrating the plant tissue. The reduction in UV-B penetration most likely caused minor damage to the plants, which continued their relatively normal developmental process without a considerable loss in final yield. Qaderi and Reid [39] reported similar results under +UV-B + CO_2_ treatment in *Brassica napus.* In contrast to our findings, Martel et al. [97] observed higher epicuticular wax in leaves of *Brassica napus* under high temperatures, and Steinmüller and Tevini [98] reported that enhanced UV-B radiation increased wax content by 23% in barley and 28% in the bean. In a study by Gonzalez et al. [99], six pea genotypes differing in their surface waxiness showed increased wax content under 6.5 kJ m^−2^ per day UV-B radiation. Higher wax content on the leaves of plants exposed to UV-B radiation indicates the importance of this chemical in plant defense mechanisms against environmental stresses. Both kale and mustard produced less wax content under high UV-B levels, pointing toward their weaker chemical defense against abiotic stresses.

The combined stress response index used in this study integrates the morphological and physiological responses, which reflect the overall sensitivity of kale and mustard to multiple stress conditions. The lowest CSRI value for both crops was observed under +T treatment, suggesting higher harmful effects of high-temperature treatment on all the parameters. The highest value for kale observed under +UV-B + CO_2_ treatment and for mustard under +T + CO_2_ treatment indicate the positive impacts of elevated CO_2_ concentrations.

## 5. Conclusions

Most current studies on plant stress response have mainly focused on the effect of individual stresses. However, combined, and sequential stress responses must be thoroughly studied to gain a meaningful understanding. The interaction of temperature stress, elevated CO_2_, and UV-B levels significantly impacted kale and mustard plants’ morphological and physiological processes. High temperature and UV-B conditions had considerable detrimental effects on most of the parameters while, under elevated CO_2_ concentration, a positive increase in all morphological and physiological traits was observed. This study recommends that varying the temperature and UV-B radiation levels in kale and mustard plants would significantly affect the growth and developmental rates and biochemistry compared to increasing the CO_2_ concentrations, which mitigates the constraining effects of temperature and UV-B stress. Farmers and researchers should, thus, attach much more importance to optimizing environmental conditions to enhance vegetable production [100,101]. Vegetables, such as kale and mustard, have been widely recommended in people’s daily diets as they provide various healthy compounds, such as antioxidants, vitamins, minerals, and dietary fiber [102]. Therefore, more research needs to be focused on the effect of multiple stresses on the nutritional quality of leafy vegetables.

## Figures and Tables

**Table 1:** The set treatments and results measured for day, night, and average temperatures, chamber [CO_2_], daytime and nighttime vapor pressure deficit (VPD), and evapotranspiration (ET) during the experimental period of each treatment on kale and mustard.

Treatments	Measured Temperature (°C)			CO_2_ (µmol mol^−1^)	VPD (kPa)		Mean ET (L H_2_O d^−1^)
	DAY	NIGHT	DAY/NIGHT	DAY	DAY	NIGHT	DAY/NIGHT
Control	24.90 ± 0.08	17.42 ± 0.03	21.56 ± 0.04	433 ± 1.92	1.35 ± 0.02	0.94 ± 0.01	11.55 ± 0.84
+CO_2_	25.13 ± 0.06	17.54 ± 0.02	21.73 ± 0.04	721.22 ± 1.27	1.33 ± 0.02	0.97 ± 0.01	11.71 ± 1.15
+T	31.31 ± 0.66	23.73 ± 0.65	27.93 ± 0.64	434.31 ± 1.30	2.30 ± 0.11	1.68 ± 0.08	12.56 ± 1.13
+UV-B	24.84 ±0.10	17.31 ± 0.03	21.48 ± 0.05	439.69 ± 1.72	1.37 ± 0.01	0.97 ± 0.01	8.48 ± 0.57
+T + CO_2_	31.69 ± 0.70	24.04 ± 0.69	28.28 ± 0.68	720.04 ± 2.49	2.75± 0.11	1.91 ± 0.08	13.79 ± 1.40
+UV-B + CO_2_	24.90 ± 0.09	17.35 ± 0.03	21.53 ± 0.05	715.98 ± 2.17	1.32 ± 0.02	0.90 ± 0.01	8.71 ± 1.01
+UV-B + T	31.32 ± 0.67	23.75 ± 0.66	27.94 ± 0.65	435.33 ± 1.27	2.74 ± 0.11	1.90 ± 0.09	11.68 ± 0.96
+UV-B + CO_2_ + T	31.34 ± 0.67	23.75 ± 0.66	27.96 ± 0.65	729.37 ±1.46	2.89 ± 0.14	1.99 ± 0.10	11.34 ± 1.21

During the experiment, the incoming daily solar radiation measured with a pyranometer (Model 4–8; The Eppley Laboratory Inc., Newport, RI, USA) outside the SPAR units ranged from 11.3 to 31.3 MJ m^2^ d^−1^ with an average value of 25.10 ± 0.82 MJ m^2^ d^−1^.

**Table 2:** The analysis of variance across the treatments of CO_2_ concentration, temperature, UV-B radiation, and two crops (kale and mustard), and their interactions on kale and mustard root and shoot growth and developmental traits, plant height (PH), mainstem leaves (LN), whole plant leaf area (LA), marketable fresh weight (MFW), aboveground dry weight (ADW), root dry weight (RDW), total plant dry weight (TDW), root/shoot ratio (RS), neoxanthin concentration (Neo), violaxanthin concentration (Viol), zeaxanthin concentration (Zea), lutein concentration (Lut), β-carotene concentration (Bcar), total xanthophyll concentration (TXan), xanthophyll cycle ratio (ZA/ZAV), chlorophyll concentration (Chl), flavonoid index (Flav), anthocyanin index (Anth), nitrogen balance index (NBI), and wax content (Wax).

Source of Variance	PH	LN	LA	MFW	ADW	RDW	TDW	RS	Neo	Viol	Zea	Lut	Bcar	TXan	ZA/ZAV	Chl	Flav	Anth	NBI	Wax
Treatment	***	***	***	***	***	***	***	***	***	***	N.S.	**	**	***	**	***	***	***	***	***
Crop	***	***	***	***	***	***	***	**	*	**	**	***	N.S.	N.S.	**	***	N.S.	***	***	***
Trt *Crop	***	***	***	**	N.S.	N.S.	N.S.	N.S.	N.S.	N.S.	N.S.	N.S.	N.S.	N.S.	N.S.	**	**	***	*	***

*** indicates significance levels, **, * and N.S., representing *p* < 0.001, *p* < 0.01, *p* < 0.05, and *p* > 0.05, respectively.

**Table 3:** Mean values and percent change for plant height (PH), leaf number (LN), leaf area (LA), marketable fresh weight (MFW), root/shoot ratio (RS), aboveground dry weight (ADW), root dry weight (RDW), and total dry weight (TDW) measured under CO_2_ concentration (control, 400 μmol mol^−1^ and + CO_2_, 720 μmol mol^−1^), elevated temperatures (25/17 °C and 35/27 °C (day/night)), and UV-B radiation (control, 0 kJ m^−2^ d^−1^ and + UV-B, 10 kJ m^−2^ d^−1^), and their interactions for kale and mustard at 40 DAS.

	Traits	Crop	Treatments							
Shoot Traits			Control	+CO_2_	+T	+UV-B	+T + CO_2_	+UV-B + CO_2_	+UV-B + T	+UV-B + CO_2_ + T
	PH (cm)	Kale	53.8	57.7 (+7%)	37.7 (−29.8%)	28.3 (−47.3%)	35.7 (−33.6%)	37.1 (−31%)	31.1 (−42%)	35.1 (−34.7%)
		Mustard	52.4	56.1 (+7%)	38.7 (−26%)	40.3 (−23%)	36.3 (−30.7%)	44 (−16%)	39.6 (−24.5%)	42.4 (−19%)
	LN (plant^−1^)	Kale	14.2	14.7 (+3%)	13 (−8.5%)	14.3 (0%)	13.8 (−3%)	15.3 (+7.8%)	13.4 (−5.4%)	14 (−1.5%)
		Mustard	32	43 (+34%)	36.2 (+13%)	20.6 (−35.6%)	50.1 (+50.6%)	28.6 (−10.7%)	30.7 (−4%)	36.7 (+14.8%)
	LA (cm^2^ plant^−1^)	Kale	1805.7	2558.7 (+39%)	1357.3 (−24.8%)	570.1 (−68.4%)	1843.6 (+2%)	1136.9 (−37%)	882.8 (−51%)	1153.1 (−36%)
		Mustard	4613.7	6220.2 (+34.8%)	3724.6 (−19%)	1934.7 (−58%)	4993.5 (+8%)	2857.1 (−38%)	2637.4 (−42.8%)	3830.9 (−17%)
	MFW (g plant^1^)	Kale	241.3	359.2 (+48.8%)	151 (−37.4%)	85.9 (−64.3%)	188.8 (−21.7%)	176.8 (−26.7%)	105.3 (−56.3%)	151.7 (37%)
		Mustard	569.2	755 (+32.6)	353 (−40%)	256 (−55%)	437.8 (−23%)	384.6 (−32.4%)	266.2 (−53%)	404.2 (−30%)
Dry weight traits	RS	Kale	0.27	0.35 (+29.6%)	0.32 (+18.5%)	0.46 (+70%)	0.28 (+3.7%)	0.51 (+88.8%)	0.31 (+14.8%)	0.37 (+37%)
		Mustard	0.27	0.35 (+29.6%)	0.32 (+18.5%)	0.36 (+33.3%)	0.23 (−14.8%)	0.38 (+40.7%)	0.33 (+22.2%)	0.28 (+3.7%)
	ADW (g plant^−1^)	Kale	21.7	37.4 (+72.1%)	17 (−21.7%)	8.9 (−59%)	22.4 (+3.2%)	19.4 (−10.8%)	10.9 (−49.4%)	17.3 (−20%)
		Mustard	38.6	57.9 (+50%)	33.2 (−14%)	21.7 (−43.6%)	40.4 (+4.7%)	33.4 (−13.3%)	26.3 (−31.8%)	38 (−1.5%)
	RDW (g plant^−1^)	Kale	6	11.7 (+95%)	5.5 (−8.5%)	4.3 (−28%)	6.4 (+6.5%)	9.6 (+60.5%)	3.3 (−45.3%)	6.3 (+5.6%)
		Mustard	10	13.9 (+39%)	10.4 (+3.9%)	7.6 (−24%)	8.9 (−10.3%)	12.6 (+25.6%)	8.7 (−12.8%)	10.4 (+4.4%)
	TDW (g plant^−1^)	Kale	27.7	49 (+76.8%)	22.5 (−19%)	13.2 (−52.3%)	28.8 (+4%)	29 (+4.6%)	14.2 (−48.5%)	23.7 (−14.5%)
		Mustard	48.6	71.8 (+47.8%)	43.6 (−10%)	29.3 (−39.6%)	49.4 (+1.6%)	45.9 (−5.3%)	35 (−28%)	48.5 (0%)

**Table 4:** Mean values and percent change for chlorophyll concentration (Chl), flavonoid index (Flav), anthocyanin index (Anth), nitrogen balance index (NBI), and wax content (Wax) under CO_2_ concentration (control, 400 μmol mol^−1^ and + CO_2_, 720 μmol mol^−1^), elevated temperatures (25/17 °C and 35/27 °C (day/night)), and UV-B radiation (control, 0 kJ m^−2^ d^−1^ and + UV-B, 10 kJ m^−2^ d^−1^), and their interactions for kale and mustard at 35 DAS.

Traits	Crop	Treatments							
		Control	+CO_2_	+T	+UV-B	+T + CO_2_	+UV-B + CO_2_	+UV-B + T	+UV-B + CO_2_ + T
Chlorophyll conc. (µg/cm^2^)	Kale	30.4	37 (+21.7%)	31.5 (+3.6%)	43.6 (+43.4%)	33.8 (+11%)	42 (+38%)	38.8 (+27.6%)	38.8 (+27.6%)
	Mustard	18.77	20.6 (+9.7%)	24 (+27.8%)	27.1 (+44.3%)	24.7 (+24.2%)	29.3 (+56%)	36.3 (+93.3%)	29.8 (+58.7%)
Flavonoid index	Kale	0.72	0.81 (+12.5%)	0.74 (+2.7%)	1.21 (+68%)	0.81 (+12.5%)	1.26 (+75%)	1.25 (+73.6%)	1.32 (+83.3%)
	Mustard	0.82	0.85 (+3.6%)	0.83 (+1.2%)	1 (+30%)	0.84 (+2.4%)	1.12 (+36.5%)	1.19 (+45%)	1.40 (+70.7%)
Anthocyanin index	Kale	0.09	0.09 (0%)	0.08 (−11%)	0.06 (−33.3%)	0.09 (0%)	0.06 (−33.3%)	0.07 (−22.2%)	0.06 (−33.3%)
	Mustard	0.14	0.13 (−7%)	0.12 (−14.2%)	0.12 (−14.2%)	0.12 (−14.2%)	0.11 (−21.4%)	0.09 (−35.7%)	0.10 (−28.5%)
NBI	Kale	42.8	46.4 (+8.4%)	46.2 (+8%)	37.7 (−11.9%)	43 (+0.5%)	34.4 (−19.6%)	31.5 (−26.4%)	30 (−30%)
	Mustard	23.4	24.6 (+5%)	31.4 (+34%)	28.5 (+21.7%)	36.5 (+60%)	27.4 (+17%)	31.8 (+35.8%)	21.9 (−6.4%)
Waxes (µg/cm^2^)	Kale	122.5	115.6 (−5.6%)	133 (+8.5%)	92.5 (−24.4%)	140.82 (+15%)	87.9 (−28.2%)	66.3 (−45.8%)	70.1 (−42.7%)
	Mustard	17.4	13 (−25.2%)	14.7 (−15.5%)	13.4 (−30%)	18.18 (+4.5%)	11 (−36.7%)	17.5 (0%)	21 (+20.6%)

**Table 5:** Mean values and percent change for neoxanthin concentration (Neo), violaxanthin concentration (Viol), zeaxanthin concentration (Zea), lutein concentration (Lut), β-carotene concentration (Bcar), total xanthophyll concentration (TXan), and xanthophyll cycle ratio (ZA/ZAV), measured under CO_2_ concentration (control, 400 μmol mol^−1^ and + CO_2_, 720 μmol mol^−1^), elevated temperatures (25/17 °C and 35/27 °C (day/night)), and UV-B radiation (control, 0 kJ m^−2^ d^−1^ and + UV-B, 10 kJ m^−2^ d^−1^), and their interactions for kale and mustard at 40 DAS.

Traits	Crop	Treatments							
		Control	+CO_2_	+T	+UV-B	+T + CO_2_	+UV-B + CO_2_	+UV-B + T	+UV-B + CO_2_ + T
Neo (µg/g dry mass)	Kale	290	355 (+22.4%)	276.8 (−4.5%)	407.7 (+40.5%)	298.5 (+3%)	372.1 (+28.3%)	371.5 (+28.1%)	397 (+36.8%)
	Mustard	273.3	256.3 (−6.2%)	246.3 (−9.8%)	370.4 (+35.5%)	269 (−1.6%)	361.2 (+32%)	378.5 (+38.4%)	319.3 (+16.8%)
Viol (µg/g dry mass)	Kale	163.7	195 (+19%)	167.5 (−2.3%)	314.4 (+92%)	150 (−8.3%)	337.8 (+106%)	276.2 (+68.8%)	242 (+47.8%)
	Mustard	323.9	217.3 (−40%)	248.8 (−23%)	327.7 (+1%)	208 (−35.7%)	274.8 (−15%)	391.2 (+20.7%)	264 (−18.4%)
Anth (µg/g dry mass	Kale	37.6	39.3 (+4.5%)	23.8 (−36.7%)	46.6 (+23.9%)	34.6 (−8%)	41.6 (+10.6%)	43.6 (+16%)	31.2 (−17%)
	Mustard	64.1	48.2 (−24.8%)	44.6 (−30.4%)	67.7 (+5.6%)	38.3 (−40%)	51.8 (−19%)	45.4 (−29%)	37.7 (−41%)
Zea (µg/g dry mass)	Kale	209.5	219.2 (+4.6%)	132.9 (−36.5%)	149.4 (−28.6%)	139.2 (−33.5%)	157.4 (−24.8%)	140.8 (−32.7%)	147.5 (−29.5%)
	Mustard	129.6	126.2 (−2.6%)	99.1 (−23.5%)	148.7 (+14.7%)	146.8 (+13.2%)	138.5 (+6.8%)	123.9 (−4.3%)	139.2 (+7.4%)
Lut (µg/g dry mass)	Kale	898.3	967.3 (+7.6%)	825 (−8%)	1093.6 (+21.7%)	679.2 (24.3%)	1105.5 (+23%)	1066.9 (+18.7%)	948.1 (−5.5%)
	Mustard	746.7	635.7 (−14.8%)	676.4 (−9.4%)	766.7 (+2.6%)	654.8 (−12.3%)	742.3 (−0.5%)	899.6 (+20.4%)	695 (−6.9%)
Bcar (µg/g dry mass)	Kale	548.4	656.7 (+19.7%)	580.5 (+5.8%)	477.6 (−13%)	439.8 (−19.8%)	710.7 (+29.5%)	766.6 (+39.7%)	636.8 (+16%)
	Mustard	649	521.1 (−19.7%)	588.2 (−9.3%)	620.9 (−4.3%)	476.6 (−26.5%)	517.7 (−20%)	745.2 (+14.8%)	568.3 (−12.4%)
Total Xanth (µg/g dry mass)	Kale	410.8	453.5 (+10.3%)	324.1 (−21%)	510.3 (+24.2%)	323.7 (−21.2%)	536.7 (+30.6%)	460.6 (+12%)	420.7 (+2.4%)
	Mustard	517.6	391.8 (−24.3%)	392.5 (−24.1%)	544 (+5.2%)	393.3 (−24%)	465 (−10%)	560.5 (+8.2%)	440.9 (−14.8%)
ZA/ZAV	Kale	0.60	0.57 (−5%)	0.48 (−20%)	0.38 (−36.6%)	0.53 (−11.6%)	0.37 (−38.3%)	0.40 (−33.3%)	0.44 (−26.6%)
	Mustard	0.37	0.44 (+18.9%)	0.36 (−2.7%)	0.41 (+10.8%)	0.47 (+27%)	0.41 (+10.8%)	0.30 (−18.9%)	0.40 (+8.1%)

## Data Availability

Not applicable.

## References

[1] Meena K.K., Sorty A.M., Bitla U.M., Choudhary K., Gupta P., Pareek A., Minhas P.S. (2017). Abiotic stress responses and microbe-mediated mitigation in plants: The omics strategies. Front. Plant Sci..

[2] Myers S.S., Smith M.R., Guth S., Golden C.D., Vaitla B., Mueller N.D., Huybers P. (2017). Climate change and global food systems: Potential impacts on food security and undernutrition. Annu. Rev. Public Health.

[3] Sinha P., Singh V.K., Bohra A., Kumar A., Reif J.C., Varshney R.K. (2021). Genomics and breeding innovations for enhancing genetic gain for climate resilience and nutrition traits. Theor. Appl. Genet..

[4] Bilal S., Shahzad R., Imran M., Jan R., Kim K.M., Lee I.J. (2020). Synergistic association of endophytic fungi enhances *Glycine max* L. resilience to combined abiotic stresses: Heavy metals, high temperature and drought stress. Ind. Crops Prod..

[5] Fawzy S., Osman A.I., Doran J., Rooney D.W. (2020). Strategies for mitigation of climate change: A review. Environ. Chem. Lett..

[6] Zandalinas S.I., Fritschi F.B., Mittler R. (2021). Global warming, climate change, and environmental pollution: Recipe for a multifactorial stress combination disaster. Trends Plant Sci..

[7] Intergovernmental Panel on Climate Change (IPCC) (2018). Global warming of 1.5 °C. An IPCC special report on the impacts of global warming of 1.5 °C above pre-industrial levels and related global greenhouse gas emission pathways. The Context Of Strengthening the Global Response to the Threat of Climate Change, Sustainable Development, and Efforts to Eradicate Poverty.

[8] Stocker T.F., Qin D., Plattner G.K., Tignor M., Allen S.K., Boschung J., Nauels A., Xia Y., Bex V., Midgley P.M. (2013). Climate Change 2013: The Physical Science Basis.

[9] Intergovernmental Panel on Climate Change (2014). Impacts, Adaptation, and Vulnerability. Part A: Global and Sectoral Aspects.

[10] Obata T., Witt S., Lisec J., Palacios-Rojas N., Florez-Sarasa I., Yousfi S., Fernie A.R. (2015). Metabolite profiles of maize leaves in drought, heat, and combined stress field trials reveal the relationship between metabolism and grain yield. Plant Physiol..

[11] Anwar K., Joshi R., Dhankher O.P., Singla-Pareek S.L., Pareek A. (2021). Elucidating the Response of Crop Plants towards Individual, Combined and Sequentially Occurring Abiotic Stresses. Int. J. Mol. Sci..

[12] Thavarajah P., Abare A., Basnagal S., Lacher C., Smith P., Combs G.F. (2016). Mineral micronutrient and prebiotic carbohydrate profiles of USA-Grown kale (*Brassica oleracea* L. var. acephala). J. Food Compos. Anal..

[13] Migliozzi M., Thavarajah D., Thavarajah P., Smith P. (2015). Lentil and kale: Complementary nutrient-rich whole food sources to combat micronutrient and calorie malnutrition. Nutrients.

[14] Pathirana I., Thavarajah P., Siva N., Wickramasinghe A.N., Smith P. (2017). Moisture deficit effects on kale (*Brassica oleracea* L. var. acephala) biomass, mineral, and low molecular weight carbohydrate concentrations. Sci. Hortic..

[15] US Department of Health and Human Services, U.S. Department of Agriculture (2015). 2015–2020 Dietary Guidelines for Americans.

[16] Rakariyatham N., Sakorn P. (2002). Biodegradation of glucosinolates in brown mustard seed meal (Brassica juncea) by Aspergillus sp. NR-4201 in liquid and solid-state cultures. Biodegradation.

[17] Cools K., Terry L.A. (2018). The effect of processing on the glucosinolate profile in mustard seed. Food Chem..

[18] Banadyga A.A. (1977). Greens or “Potherbs”—Chard, Collards, Kale, Mustard, Spinach.

[19] Deryng D., Conway D., Ramankutty N., Price J., Warren R. (2014). Global crop yield response to extreme heat stress under multiple climate change futures. Environ. Res. Lett..

[20] Ugarte C., Calderini D.F., Slafer G.A. (2007). Grain weight and grain number responsiveness to pre-anthesis temperature in wheat, barley and triticale. Field Crops Res..

[21] Asseng S., Foster I.A.N., Turner N.C. (2011). The impact of temperature variability on wheat yields. Glob. Change Biol..

[22] Allen L.H., Boote K.J., Reddy K.R., Hodges H.F. (2000). Crop ecosystem responses to climatic change: Soybean. Climate Change and Global Crop Productivity.

[23] Reddy K.R., Hodges H.F. (2000). Climate Change and Global Crop Productivity.

[24] Ainsworth E.A., Davey P.A., Bernacchi C.J., Dermody O.C., Heaton E.A., Moore D.J., Long S.P. (2002). A meta-analysis of elevated [CO_2_] effects on soybean (*Glycine max*) physiology, growth and yield. Glob. Change Biol..

[25] Kakani V.G., Reddy K.R., Zhao D., Mohammed A.R. (2003). Effects of ultraviolet-B radiation on cotton (*Gossypium hirsutum* L.) morphology and anatomy. Ann. Bot..

[26] Kakani V.G., Reddy K.R., Zhao D., Sailaja K. (2003). Field crop responses to ultraviolet-B radiation: A review. Agric. For. Meteorol..

[27] Li Y., He L., Zu Y. (2010). Intraspecific variation in sensitivity to ultraviolet-B radiation in endogenous hormones and photosynthetic characteristics of 10 wheat cultivars grown under field conditions. S. Afr. J. Bot..

[28] Reddy K.R., Singh S.K., Koti S., Kakani V.G., Zhao D., Gao W., Reddy V.R. (2013). Quantifying corn growth and physiological responses to ultraviolet-B radiation for modeling. Agron. J..

[29] Singh S.K., Reddy K.R., Reddy V.R., Gao W. (2014). Maize growth and developmental responses to temperature and ultraviolet-B radiation interaction. Photosynthetica.

[30] Kataria S., Jajoo A., Guruprasad K.N. (2014). Impact of increasing Ultraviolet-B (UV-B) radiation on photosynthetic processes. J. Photochem. Photobiol. B Biol..

[31] Allen L.H., Boote K.J., Jones J.W., Jones P.H., Pickering N.B., Baker J.T., Prasad P.V.V. (2020). Sunlit, controlled-environment chambers are essential for comparing plant responses to various climates. Agron. J..

[32] Reddy K.R., Read J.J., McKinion J.M. (2001). Soil-Plant-Atmosphere-Research (SPAR) facility: A tool for plant research and modeling. Biotronics.

[33] Hewitt E.J. (1952). Sand and Water Culture Methods Used in the Study of Plant Nutrition.

[34] Ebercon A., Blum A., Jordan W.R. (1977). A Rapid Colorimetric Method for Epicuticular Wax Contest of Sorghum Leaves. Crop Sci..

[35] Kopsell D.A., Kopsell D.E., Curran-Celentano J. (2007). Carotenoid pigments in kale are influenced by nitrogen concentration and form. J. Sci. Food Agric..

[36] Kopsell D.A., Kopsell D.E., Lefsrud M.G., Curran-Celentano J., Dukach L.E. (2004). Variation in Lutein, β-carotene, and Chlorophyll Concentrations among *Brassica oleracea* Cultigens and Seasons. HortScience.

[37] Barickman T.C., Kopsell D.A., Sams C.E. (2016). Abscisic Acid Impacts Tomato Carotenoids, Soluble Sugars, and Organic Acids. HortScience.

[38] Koti S., Reddy K.R., Reddy V.R., Kakani V.G., Zhao D. (2005). Interactive effects of carbon dioxide, temperature, and ultraviolet-B radiation on soybean (*Glycine max* L.) flower and pollen morphology, pollen production, germination, and tube lengths. J. Exp. Bot..

[39] Qaderi M.M., Reid D.M. (2005). Growth and physiological responses of canola (*Brassica napus*) to UV-B and CO_2_ under controlled environment conditions. Physiol. Plant..

[40] Rodríguez-Calzada T., Qian M., Strid Å., Neugart S., Schreiner M., Torres-Pacheco I., Guevara-González R.G. (2019). Effect of UV-B radiation on morphology, phenolic compound production, gene expression, and subsequent drought stress responses in chili pepper (*Capsicum annuum* L.). Plant Physiol. Biochem..

[41] Son K.H., Ide M., Goto E. (2020). Growth characteristics and phytochemicals of canola (*Brassica napus*) grown under U.V. radiation and low root zone temperature in a controlled environment. Hortic. Environ. Biotechnol..

[42] Lercari B., Sodi F., Di Paola M.L. (1990). Photomorphogenic responses to U.V. radiation: Involvement of phytochrome and U.V. photoreceptors in the control of hypocotyl elongation in *Lycopersicon esculentum*. Physiol. Plant..

[43] Corlett J.E., Stephen J., Jones H.G., Woodfin R., Mepsted R., Paul N.D., Lumsden P.J. (1997). Assessing the impact of UV-B radiation on the growth and yield of field crops. Plants and UV-B: Responses to Environmental Change.

[44] Conner J.K., Zangori L.A. (1997). A garden study of the effects of ultraviolet-B radiation on pollination success and lifetime female fitness in Brassica. Oecologia.

[45] Prasad P.V.V., Staggenborg S.A., Ristic Z., Ahuja L.H., Saseendran S.A. (2008). Impacts of drought and/or heat stress on physiological, developmental, growth, and yield processes of crop plants. Response of Crops to Limited Water: Understanding and Modeling Water Stress Effects on Plant Growth Processes.

[46] Rodríguez M., Canales E., Borrás-Hidalgo O. (2005). Molecular aspects of abiotic stress in plants. Biotecnol. Apl..

[47] Hasanuzzaman M., Nahar K., Alam M., Roychowdhury R., Fujita M. (2013). Physiological, biochemical, and molecular mechanisms of heat stress tolerance in plants. Int. J. Mol. Sci..

[48] Chauhan J.S., Meena M.L., Saini M.K., Meena D.R., Singh M., Meena S.S., Singh K.H. Heat stress effects on morph-physiological characters of Indian Mustard (*Brassica juncea* L.). Proceedings of the 16th Australian Research Assembly on Brassicas.

[49] Schaarschmidt S., Lawas L.M.F., Glaubitz U., Li X., Erban A., Kopka J., Zuther E. (2020). Season affects yield and metabolic profiles of rice (*Oryza sativa*) under high night temperature stress in the field. Int. J. Mol. Sci..

[50] Kosova K., Vitamvas P., Prasil I.T., Renaut J. (2011). Plant proteome changes under abiotic stress—Contribution of proteomics studies to understanding plant stress response. J. Proteom..

[51] Akter N., Islam M.R. (2017). Heat stress effects and management in wheat. A review. Agron. Sustain. Dev..

[52] Qaderi M.M., Kurepin L.V., Reid D.M. (2006). Growth and physiological responses of canola (*Brassica napus*) to three components of global climate change: Temperature, carbon dioxide and drought. Physiol. Plant..

[53] Qaderi M.M., Basraon N.K., Chinnappa C.C., Reid D.M. (2010). Combined effects of temperature, ultraviolet-B radiation, and watering regime on growth and physiological processes in canola *(Brassica napus*) seedlings. Int. J. Plant Sci..

[54] Pérez M.L., Prado F.E., González J.A. (2015). Effects of ultraviolet B (UV-B) on different varieties of quinoa. I. Effects on morphology under controlled conditions. Bol. Soc. Argent. Bot..

[55] Hinojosa L., González J.A., Barrios-Masias F.H., Fuentes F., Murphy K.M. (2018). Quinoa abiotic stress responses: A review. Plants.

[56] Raghuvanshi R., Sharma R.K. (2016). Response of two cultivars of *Phaseolus vulgaris* L. (French beans) plants exposed to enhanced UV-B radiation under mountain ecosystem. Environ. Sci. Pollut. Res..

[57] Wargent J.J., Gegas V.C., Jenkins G.I., Doonan J.H., Paul N.D. (2009). UVR8 in Arabidopsis thaliana regulates multiple aspects of cellular differentiation during leaf development in response to ultraviolet B radiation. New Phytol..

[58] Nogués S., Allen D.J., Morison J.I., Baker N.R. (1999). Characterization of stomatal closure caused by ultraviolet-B radiation. Plant Physiol..

[59] Nedunchezhian N., Kulandaivelu G. (1997). Changes induced by ultraviolet-B (280–320 nm) to vegetative growth and photosynthetic characteristics in filed grown *Vigna unguiculate* L.. Plant Sci..

[60] Cechin I., Fumis T.D.F., Dokkedal A.L. (2007). Growth and physiological responses of sunflower plants exposed to ultraviolet-B radiation. Ciênc. Rural.

[61] Gao W., Zheng Y.F., Slusser J.R., Heisler G.M., Grant R.H., Xu J.Q., He D.L. (2004). Effects of supplementary ultraviolet-B irradiance on maize yield and qualities: A field experiment. J. Photochem. Photobiol..

[62] Rajabbeigi E., Eichholz I., Beesk N., Ulrichs C., Kroh L.W., Rohn S., Huyskens-Keil S. (2013). Interaction of drought stress and UV-B radiation-impact on biomass production and flavonoid metabolism in lettuce (*Lactuca sativa* L.). J. Appl. Bot. Food Qual..

[63] Balasooriya H.N., Dassanayake K.B., Seneweera S., Ajlouni S. (2018). Interaction of elevated carbon dioxide and temperature on strawberry (Fragaria× ananassa) growth and fruit yield. Int. J. Agric. Biosyst. Eng..

[64] Poulson M.E., Boeger M.R.T., Donahue R.A. (2006). Response of photosynthesis to high light and drought for Arabidopsis thaliana grown under a UV-B enhanced light regime. Photosynth. Res..

[65] Koti S., Reddy K.R., Kakani V.G., Zhao D., Gao W. (2007). Effects of carbon dioxide, temperature and ultraviolet-B radiation and their interactions on soybean (*Glycine max* L.) growth and development. Environ. Exp. Bot..

[66] Wijewardana C., Henry W.B., Gao W., Reddy K.R. (2016). Interactive effects on CO_2_, drought, and ultraviolet-B radiation on maize growth and development. J. Photochem. Photobiol..

[67] Wanzeler R.B., Zanetti L.V., Fantinato D.E., Gama V.N., Arrivabene H.P., de Almeida Leite I.T., Milanez C.R.D. (2019). How does UV-B radiation affect the initial growth of common bean (*Phaseolus vulgaris* L.)? Physiological and structural aspects. Braz. J. Dev..

[68] Chen Z., Gao W., Reddy K.R., Chen M., Taduri S., Meyers S.L., Shankle M.W. (2020). Ultraviolet (U.V.) B effects on growth and yield of three contrasting sweet potato cultivars. Photosynthetica.

[69] Barickman T.C., Olorunwa O.J., Sehgal A., Walne C.H., Reddy K.R., Gao W. (2021). Interactive impacts of temperature and elevated CO_2_ on Basil (*Ocimum basilicum* L.) root and shoot morphology and growth. Horticulturae.

[70] Sakalauskaite J., Viškelis P., Duchovskis P., Dambrauskiene E., Sakalauskiene S., Samuoliene G., Brazaityte A. (2012). Supplementary UV-B irradiation effects on basil (*Ocimum basilicum* L.) growth and phytochemical properties. J. Food Agric. Environ..

[71] Wang W., Cai C., He J., Gu J., Zhu G., Zhang W., Liu G. (2020). Yield, dry matter distribution and photosynthetic characteristics of rice under elevated CO_2_ and increased temperature conditions. Field Crops Res..

[72] Yuan L., Tang L., Zhu S., Hou J., Chen G., Liu F., Wang C. (2017). Influence of heat stress on leaf morphology and nitro gen–carbohydrate metabolisms in two wucai (*Brassica campestris* L.) genotypes. Acta Soc. Bot. Pol..

[73] Boeger M.R.T., Poulson M. (2006). Effects of ultraviolet-B radiation on leaf morphology of *Arabidopsis thaliana* (L.) Heynh. (Brassicaceae). Acta Bot. Bras..

[74] Karvansara P.R., Razavi S.M. (2019). Physiological and biochemical responses of sugar beet (*Beta vulgaris* L.) to ultraviolet-B radiation. PeerJ.

[75] Al-Oudat M., Baydoun S.A., Mohammad A. (1998). Effects of enhanced UV-B on growth and yield of two Syrian crops wheat (*Triticum durum* var. Horani) and broad beans (*Vicia faba*) under field conditions. Environ. Exp. Bot..

[76] Zhang X.R., Chen Y.H., Guo Q.S., Wang W.M., Liu L., Fan J., Li C. (2017). Short-term UV-B radiation effects on morphology, physiological traits and accumulation of bioactive compounds in *Prunella vulgaris* L.. J. Plant Interact..

[77] Hideg É., Jansen M.A., Strid Å. (2013). UV-B exposure, ROS, and stress: Inseparable companions or loosely linked associates?. Trends Plant Sci..

[78] Ziska L.H., Teramura A.H., Sullivan J.H., McCoy A. (1993). Influence of ultraviolet-B (UV-B) radiation on photosynthetic and growth characteristics in field-grown cassava (*Manihot esculentum* Crantz). Plant Cell Environ..

[79] Idso S.B., Kimball B.A., Mauney J.R. (1988). Effects of atmospheric CO_2_ enrichment on root: Shoot ratios of carrot, radish, cotton and soybean. Agric. Ecosyst. Environ..

[80] Yang X. (1993). Plants and Microclimate: A Quantitative Approach to Environmental Plant Physiology. Agric. Forest Meteorol..

[81] Shah N.H., Paulsen G.M. (2003). Interaction of drought and high temperature on photosynthesis and grain-filling of wheat. Plant Soil.

[82] Angadi S.V., Cutforth H.W., Miller P.R., McConkey B.G., Entz M.H., Brandt S.A., Volkmar K.M. (2000). Response of three Brassica species to high temperature stress during reproductive growth. Can. J. Plant Sci..

[83] Rodríguez V.M., Soengas P., Alonso-Villaverde V., Sotelo T., Cartea M.E., Velasco P. (2015). Effect of temperature stress on the early vegetative development of *Brassica oleracea* L.. BMC Plant Biol..

[84] Choi E.Y., Seo T.C., Lee S.G., Cho I.H., Stangoulis J. (2011). Growth and physiological responses of Chinese cabbage and radish to long-term exposure to elevated carbon dioxide and temperature. Hortic. Environ. Biotechnol..

[85] Hinojosa L., Matanguihan J.B., Murphy K.M. (2019). Effect of high temperature on pollen morphology, plant growth and seed yield in quinoa (*Chenopodium quinoa* Willd.). J. Agron. Crop Sci..

[86] Sun P., Mantri N., Lou H., Hu Y., Sun D., Zhu Y., Lu H. (2012). Effects of elevated CO_2_ and temperature on yield and fruit quality of strawberry (*Fragaria× ananassa* Duch.) at two levels of nitrogen application. PLoS ONE.

[87] Kumari M., Verma S.C., Bhardwaj S.K. (2019). Effect of elevated CO_2_ and temperature on crop growth and yield attributes of bell pepper (*Capsicum annuum* L.). J. Agrometeorol..

[88] Rangaswamy T.C., Sridhara S., Ramesh N., Gopakkali P., El-Ansary D.O., Mahmoud E.A., Abdel-Hamid A.M. (2021). Assessing the impact of higher levels of CO_2_ and temperature and their interactions on tomato (*Solanum lycopersicum* L.). Plants.

[89] Zhou R., Yu X., Li X., Mendanha dos Santos T., Rosenqvist E., Ottosen C.O. (2020). Combined high light and heat stress induced complex response in tomato with better leaf cooling after heat priming. Plant Physiol. Biochem..

[90] Barickman T.C., Olorunwa O.J., Sehgal A., Walne C.H., Reddy K.R., Gao W. (2021). Yield, Physiological Performance, and Phytochemistry of Basil (*Ocimum basilicum* L.) under Temperature Stress and Elevated CO_2_ Concentrations. Plants.

[91] Yoon H.I., Kim D., Son J.E. (2020). Spatial and temporal bioactive compound contents and chlorophyll fluorescence of kale (*Brassica oleracea* L.) under UV-B exposure near harvest time in controlled environments. Photochem. Photobiol..

[92] Pourcel L., Routaboul J.M., Cheynier V., Lepiniec L., Debeaujon I. (2007). Flavonoid oxidation in plants: From biochemical properties to physiological functions. Trends Plant Sci..

[93] Olsson L.C., Veit M., Bornman J.F. (1999). Epidermal transmittance and phenolic composition in leaves of atrazine-tolerant and atrazine-sensitive cultivars of *Brassica napus* grown under enhanced UV-B radiation. Physiol. Plant..

[94] Cartelat A., Cerovic Z.G., Goulas Y., Meyer S., Lelarge C., Prioul J.L., Barbottin A., Jeuffroy M.H., Gate P., Agati G. (2005). Optically assessed contents of leaf polyphenolics and chlorophyll as indicators of nitrogen deficiency in wheat (*Triticum aestivum* L.). Field Crops Res..

[95] Oliveira A.F., Rais F., Dettori I., Azzena M., Nieddu G. (2019). UV light acclimation capacity of leaf photosynthetic and photochemical behaviour in near-isohydric and anisohydric grapevines in hot and dry environments. S. Afr. J. Enol. Vitic..

[96] Pieristè M., Chauvat M., Kotilainen T.K., Jones A.G., Aubert M., Robson M., Forey E. (2019). Solar UV-A radiation and blue light enhance tree leaf litter decomposition in a temperate forest. Oecologia.

[97] Martel A.B., Taylor A.E., Qaderi M.M. (2020). Individual and interactive effects of temperature and light intensity on canola growth, physiological characteristics and methane emissions. Plant Physiol. Biochem..

[98] Steinmüller D., Tevini M. (1985). Action of ultraviolet radiation (UV-B) upon cuticular waxes in some crop plants. Planta.

[99] Gonzalez R., Paul N.D., Percy K., Ambrose M., McLaughlin C.K., Barnes J.D., Wellburn A.R. (1996). Responses to ultraviolet-B radiation (280–315 nm) of pea (*Pisum sativum*) lines differing in leaf surface wax. Physiol. Plant..

[100] Dong J., Gruda N., Lam S.K., Li X., Duan Z. (2018). Effects of elevated CO_2_ on nutritional quality of vegetables: A review. Front. Plant Sci..

[101] Gruda N., Bisbis M., Tanny J. (2019). Impacts of protected vegetable cultivation on climate change and adaptation strategies for cleaner production–a review. J. Clean. Prod..

[102] Slavin J.L., Lloyd B. (2012). Health benefits of fruits and vegetables. Adv. Nutr..

